# Identification of novel serum peptide biomarkers for high-altitude adaptation: a comparative approach

**DOI:** 10.1038/srep25489

**Published:** 2016-05-06

**Authors:** Juan Yang, Wenhua Li, Siyuan Liu, Dongya Yuan, Yijiao Guo, Cheng Jia, Tusheng Song, Chen Huang

**Affiliations:** 1 Department of Cell Biology and Genetics, School of Basic Medical Sciences, Xi’an Jiaotong University Health Science Center, Xi’an 710061, P. R. China; 2 Key Laboratory of Environment and Genes Related to Diseases (Xi’an Jiaotong University), Ministry of Education of China, Xi’an 710061, P. R. China; 3 Xizang Minzu University Medical School, Xianyang 712082, P. R. China

## Abstract

We aimed to identify serum biomarkers for screening individuals who could adapt to high-altitude hypoxia at sea level. HHA (high-altitude hypoxia acclimated; *n* = 48) and HHI (high-altitude hypoxia illness; *n* = 48) groups were distinguished at high altitude, routine blood tests were performed for both groups at high altitude and at sea level. Serum biomarkers were identified by comparing serum peptidome profiling between HHI and HHA groups collected at sea level. Routine blood tests revealed the concentration of hemoglobin and red blood cells were significantly higher in HHI than in HHA at high altitude. Serum peptidome profiling showed that ten significantly differentially expressed peaks between HHA and HHI at sea level. Three potential serum peptide peaks (m/z values: 1061.91, 1088.33, 4057.63) were further sequence identified as regions of the inter-α trypsin inhibitor heavy chain H4 fragment (ITIH4 347–356), regions of the inter-α trypsin inhibitor heavy chain H1 fragment (ITIH1 205–214), and isoform 1 of fibrinogen α chain precursor (FGA 588–624). Expression of their full proteins was also tested by ELISA in HHA and HHI samples collected at sea level. Our study provided a novel approach for identifying potential biomarkers for screening people at sea level who can adapt to high altitudes.

The Qinghai-Tibetan plateau is the highest and largest plateau in the world. The Tibetans on the plateau reside permanently at altitudes of 3,000 to 4,500 meters^1^. Each year, a large number of people flock to the Qinghai-Tibetan plateau for outdoor activities, such as trekking, backpacking, mountain climbing, and travel. However, acute exposure to high altitude hypoxia can cause pathophysiological changes that manifest as a spectrum of disorders, ranging from the relatively benign high altitude headache to life-threatening high altitude cerebral edema^2^,^3^. High-altitude hypoxia illness (HHI) is a disease spectrum related to hypobaric hypoxia and its consequences. It includes acute mountain sickness, high-altitude cerebral edema, and high-altitude pulmonary edema^3^,^4^.

Medical problems at high altitude largely stem from hypoxia along with risks of dehydration, accidental injury, cold injury, weight loss, and psychological stress under such an environment in lowland sojourners who travel to high altitudes^5^. It is reported that ~25% of travelers develop HHI at an altitude of 2,500 meters^6^. While susceptible individuals may develop HHI at altitudes >1,371 meters^7^, most people can go up to 2,500 meters with minimal effect^8^. Why can some people tolerate high altitude and acclimatize themselves to high-altitude hypoxia (high-altitude hypoxia acclimated; HHA), while others are susceptibility to HHI? There are no specific factors, such as gender and physical condition, that correlate with susceptibility to altitude illness (http://www.thefullwiki.org/Altitude illness). Given that HHA individuals can acclimatize themselves to high-altitude hypoxia, and HHI individuals develop illness at high altitude, there should be a genetic difference between the groups at sea level.

Until now, research on high-altitude illness has mainly focused on the genetic or biochemical differences between high-altitude residents such as Tibetans and sea-level residents^9^,^10^, the physiological difference between individuals at sea level and high altitude^11^, or the physiological or other differences between HHA and HHI individuals^12^. There have been few research on high-altitude adaptation in lowland dwellers. It has become an attractive approach to search for novel serum biomarkers in biological fluids of HHA serum samples at using peptide profiling^13^,^14^. Recent advances in peptidome techniques make it possible to uncover plasma protein expression profiles in HHA and HHI individuals at sea level, and will provide a better insight into the mechanisms involved in functional adaptations of cells, tissues and organs, as well as the whole organism in the high-altitude hypoxic environment^15^,^16^. Indeed, potential serum biomarkers could be found among the specific peptides that are up- or down-regulated in serum peptidome profiling in HHA samples compared with HHI group.

In the current study, we distinguished HHA and HHI individuals at high altitudes, and then we performed routine blood tests for HHA and HHI samples at high altitudes as well as at sea level, followed by magnetic-bead-based purification combined with matrix - assisted laser desorption ionization time – of - flight mass spectrometry (MALDI - TOF MS) for serum peptidome profiling of serum samples collected at sea level. By comparing the serum peptidome profiles generated, differentially expressed peaks were listed as potential biomarkers for HHA. The potential serum biomarkers were then further identified by liquid chromatography- electrospray ionization tandem MS (LC-ESI-MS/MS), and the identified proteins were validated by ELISA (Fig. 1).

## Results

### Routine blood tests of HHA and HHI groups

**Figure 2:**
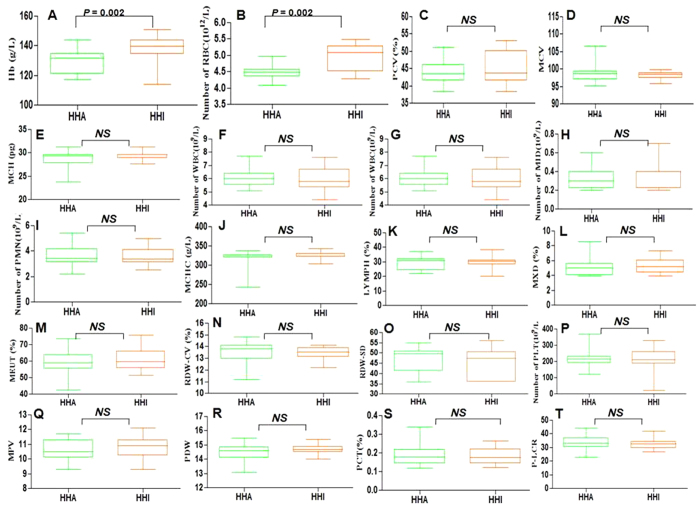
The routine blood test result for HHI and HHA group at high altitude. (**A**) The concentration of Hb in HHI was significantly higher than in HHA group. (**B**) The count of red blood cell in HHI was significantly higher than in HHA. (**C–T**) The un-changed or not significant results between HHA and HHI group, the corresponding indexes including PCV, MCV, MCH, the count of WBC, LYM, MID, PMN, PLT as well as MCHC, LYMPH, MXD, NEUT, RDW-CV, RDW-SD, MPV, PDW, PCT and P-LCR.

Concentration of hemoglobin was significantly higher in the HHI (mean value, 138.5 ±  1.67 g/L) than HHA (mean value, 129.1 ±  1.60 g/L) group (P =  0.002) at high altitude. RBC was also significantly higher in the HHI (mean value, 4.96 ×  10^12^/L) group than HHA (mean value, 4.47 ×  10^12^/L) group (P =  0.002) at high altitude (Table 1, Fig. 2). The mean values for PCV, MCV, MCH, WBC, LYM, PMN, PLT, MCHC, LYMPH, RDW-CV, RDW-SD, MPV, PDW, PCT and P-LCR did not differ significantly between the HHA and HHI at high altitude (Table 1, Fig. 2). While, it revealed that there have been no significant differences between HHA and HHI at sea level, and the results of all indexes were tested similarly, including hemoglobin content (HHI *vs* HHA, 107.8 ±  5.13 *vs* 107.7 ±  5.83) and RBC (HHI *vs* HHA, 4.62 ×  10^12^ *vs* 4.54 ×  10^12^) (Table S1).

### Serum peptidome profiles of HHA and HHI groups at sea level

**Figure 3:**
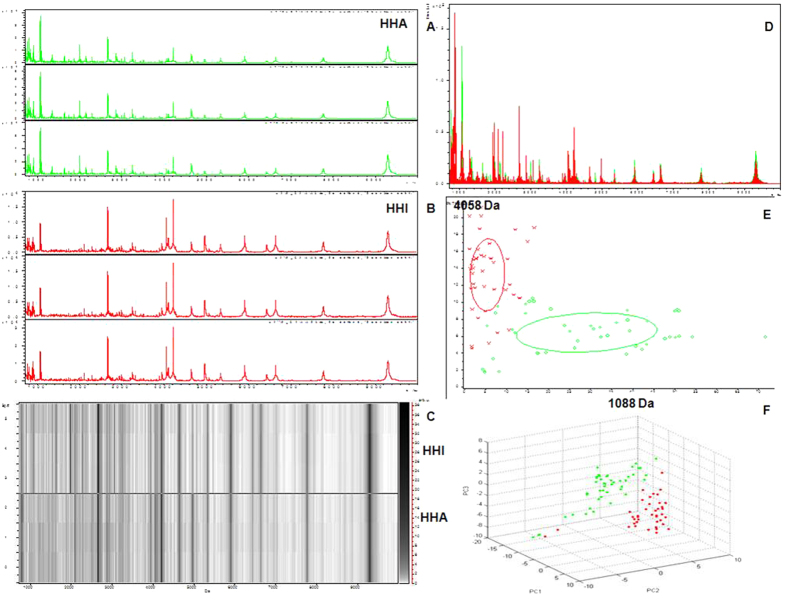
Reproducibility of mass spectra generated in individuals from HHI and HHA groups and comparative analysis of serum peptidome profiling between HHA and HHI samples collected at sea level. (**A**) Representative mass spectra of a HHA (green) at sea level, in the mass range from 1 to 10 kDa showing low variability between replicates of the sample. (**B**) Representative mass spectra of one HHI (red) sample at sea level, in the mass range from 1 to 10 kDa showing low variability between replicates of this sample. (**C**) Gel view of mass spectra from a HHA sample and a HHI sample at sea level in the mass range from 1 to 10 kDa, showing low variability between replicates of each sample. (**D**) Overall sum of the spectra in the mass range from 1 to 10 kDa obtained from all HHI group (red) and HHA samples (green) at sea level. (**E**) Bivariate plot of HHI group (red) and HHA samples (green) in the component analysis with the most differentiated two peaks (m/z: 1088, 4058). (**F**) 3D plot of HHI group (red) and HHA samples (green) after subgroup separation in the component analysis.

The reproducibility and stability of the mass spectra were evaluated in triplicate samples and showed closely reproducible peaks (Fig. 3A–C). The serum peptidome profiles of the HHA and HHI groups at sea level were compared. Fractionation of serum samples by MB-WCX and MALDI-TOF MS showed that the HHI (red) and HHA (green) group had peptidome profiles from 1 to 10 kDa (Fig. 3). Within this mass range, some differentially expressed peaks between HHI and HHA could be detected. The sample distribution chart of the HHA and HHI groups showed few overlapping areas that accurately distinguished the two groups (Fig. 3E,F).

### Peak selection and model testing

There were up to 114 different peaks identified between the HHA and HHI groups, and 78 of them were significant (P <  0.05). The 10 most significantly differentiated peaks are listed in Table 2 (P <  0.0001, fold changes > 2 or < 0.5, based on the Wilcoxon rank sum test). Among these 10 peaks, two peaks (m/z : 4057.63, m/z: 4969.09) were down-regulated in serum collected from HHA group at sea level. Another eight peaks were up-regulated in serum samples from HHA group compared with the HHI group at sea level (Table 2).

**Figure 4:**
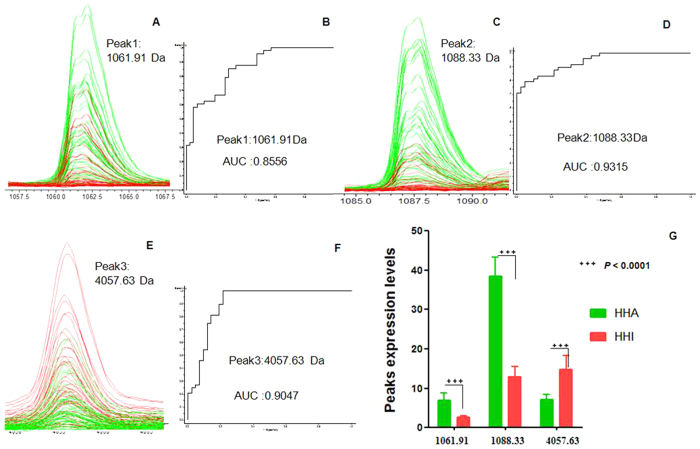
Representative spectra of three potential biomarker peaks in HHA group at sea level. (**A,C,E**) Comparison of the spectra of three peaks in the HHI group (red) and HHA samples (green). (**B,D,F)** ROC curves for three selected peaks with their AUC values. (**G**) Average expression levels of three selected peaks in HHI group (red) and HHA samples (green) and their P values. Values are expressed as the mean ±  SD.

ClinProtTools analysis of the two groups (from serum samples collected at sea level) based on the GA algorithm model showed that HHA group could be discriminated from the HHI group with 83.33% sensitivity and 86.88% specificity. The three potential serum biomarkers (Tables 2, 3) used in the GA model included one down - regulated peak (m/z values: 4057.63) and two up-regulated peaks (m/z values: 1088.33, 1061.91) in the HHA group. Comparison of the spectra of these three peaks between the HHI (red) and HHA (green) groups and their receiving operating characteristic (ROC) curves are shown in Fig. 4. The area under the curve (AUC) of these three peaks were 0.8556 (Peak 1, m/z: 1061.91), 0.9315 (Peak 2, m/z: 1088.33), and 0.9047 (Peak 3, m/z: 4057.63) (Fig. 4).

### Identification of HHA serum biomarkers at sea level

The three peptide peaks (Fig. 4, m/z values: 1061.91, 1088.33, 4057.63) were further sequenced and identified using LC-ESI-MS/MS as well as the Uniprot database. MS/MS fragmentation of these three peptides identified the relevant peptide sequences listed in Table 3. MS/MS fragmentation of these two peptides identified the sequences SEMVVAGKLQ (Fig. S1), LDAQASFLPK (Fig. S2) and SYNRGDSTFESKSYKMADEAGSEADHEGTHSTKRGHA (Fig. S3). These three peptide sequences were further identified as regions ITIH4 347–356 (m/z: 1061.91), ITIH1 205–214 (m/z: 1088.33), and FGA 588–624 (m/z: 4057.73) (Table 3).

### Full protein expression of ITIH1, ITIH4 and FGA in HHA and HHI groups at sea level

**Figure 5:**
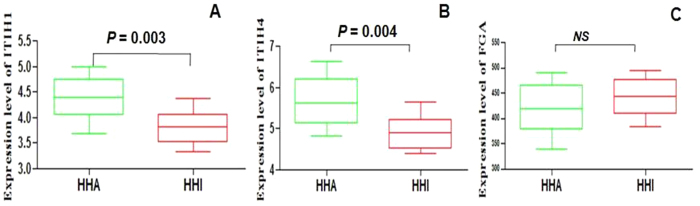
ELISA analysis of ITIH1, ITIH4, FGA in the HHI and HHA group. For all (**A–C**) y-axis represent protein expression levels (ng/mL) in sera of different groups. Statistical analysis was performed with Prism 5.0 software. All data are represented as mean ±  SD, for each group, P <  0.05 versus controls.

To screen for serum biomarkers of HHA and determine the expression level of ITIH1, ITIH4 and FGA at sea level, serum concentrations were examined by ELISA in 96 samples from the HHA and HHI groups. Serum concentrations of ITIH1, ITIH4 and FGA in the two groups are shown in Table 4 and Fig. 5.

The mean concentration of ITIH1 was 4.49 ±  0.12 ng/mL (range, 3.69–5.0 ng/mL) in the HHA group, and 3.61 ±  0.09 ng/mL (range, 3.32–4.37 ng/mL) in HHI group (P =  0.003). These results indicated that ITIH1 was expressed at significantly higher levels in HHA group (Fig. 5) and showed the same tendency as the peptides (m/z 1061.91). The mean concentration of ITIH4 was 5.67 ±  0.17 ng/mL (range, 4.82–6.64 ng/mL) in the HHA group, and 4.92 ±  0.12 ng/mL (range, 4.38–5.65 ng/mL) in HHI group (P =  0.003). These results indicated that ITIH4 was expressed at significantly higher levels in HHA group (Fig. 5) and showed the same tendency as the peptides (m/z 1088.33).

Serum concentration of FGA was 400.5 ±  13.95 ng/mL (range, 338.5–471.0 ng/mL) in the HHA group, and 446.6 ±  1.23 ng/mL (range, 395.6–493.8 ng/mL) in HHI group. These results indicated that FGA was expressed at lower levels in HHA group (Fig. 5) and showed the same tendency as the peptides (m/z 1061.91), while the difference was not significant (P >  0.05).

## Discussion

High-altitude adaptation is a classic area of research in the field of biological anthropology, and most studies have focused on the physiological effects of hypobaric hypoxia on high-altitude populations. There have been few research on high-altitude adaptation in lowland dwellers. It is of particular interest to establish why some but not all lowland people can adapt to high-altitude hypoxia. The present study is believed to be the first analysis of serum peptidome in HHA and HHI groups at sea level. We demonstrated the feasibility and safety of using serum peptidome to reveal the serum biomarkers for HHA at sea level. In addition, three potential serum peptide biomarkers were verified using ELISA, by full protein expression in the HHA and HHI groups at sea level. These peptide biomarkers and their full proteins may play a role in high-altitude adaption.

Routine blood tests revealed that the concentration of hemoglobin and RBC in HHI samples were both significantly higher than in the HHA samples at high altitude(Table 1, Fig. 2). PCV was also higher but not significantly (P >  0.05). Results for other indexes were similar in the HHI and HHA groups (Table 1, Fig. 2). While at sea level, the results were similar in HHI and HHA, including hemoglobin and RBC (Table S1), moreover, the hemoglobin contents in both groups at sea level were less than which at high altitude. Lowland mammals that are not genetically adapted to environmental hypoxia typically respond to chronic oxygen deprivation with increased erythropoietic activity, resulting in a correlative increase in hemoglobin concentration, hematocrit or RBC^17^. A moderate increase in hemoglobin concentration can be propitious to increase blood oxygen-carrying capacity and improve tissue oxygenation, which must be closer to the optimal values^18^,^19^. However, an excessive increase in hemoglobin increases blood viscosity and peripheral vascular resistance that can compromise cardiac output, and also can damage brain tissue as well as the cerebral vascular endothelial cells^19^,^20^,^21^,^22^.

In the serum peptidome study, we identified 10 differentiated expression peaks for distinguishing HHA from HHI group at sea level. Two peaks of 10 (m/z: 4057.63, 4969.09) were down-regulated in HHA group at sea level, while the remaining eight were up-regulated in HHA group at sea level. ClinProTools provided predictive models for HHA versus HHI in their serum samples at sea level, and the mean cross-validation and recognition capacities had 83.33% sensitivity and 86.88% specificity. The three potential serum biomarkers for HHA (samples at sea level) were identified as ITIH4 347–356 (Peak 1, m/z: 1061.91), ITIH1 205–214 (Peak 2, m/z: 1088.33), and FGA 588–624 (Peak 3, m/z: 4057.73). Due to there have been no antibodies to the peptides identified as potential biomarkers in this study, the expression levels of their full proteins were verified in HHA and HHI by ELISA.

Two of the identified serum biomarkers for HHA at sea level, ITIH4 347–356 (Peak 1, m/z: 1061.91) and ITIH1 205–214 (Peak 2, m/z: 1088.33) were both up-regulated in HHA samples at sea level, and their full proteins ITIH4 and ITIH1 were also tested by ELISA for their expression levels in HHI and HHA groups. ELISA showed that the full proteins were both up-regulated in HHA samples at sea level, which showed the same tendency as the peptide biomarkers. ITIH4 is a plasma glycoprotein with a relative molecular mass of 120 kDa, which acts as an acute-phase protein in several species and is expressed mainly in the liver^23^,^24^. ITIH4 is present in plasma as a single-chain protein. ITIH4 is highly sensitive to plasma kallikrein and is proposed to be a potential precursor for plasma kallikrein-induced bioactive peptides^25^. ITIH1 is one of the heavy chains of a serine protease inhibitor that may serve to carry hyaluronan in plasma, and plays a role in inflammation and carcinogenesis^26^. Some research suggested that ITIH1 is related to the incidence of bipolar disorder^27^ and human solid tumors^26^. ITIH1 and ITIH4 are expressed abundantly in the liver and contain a putative binding site for hyaluronic acid, which is a ubiquitous component of the extracellular matrix (ECM)^28^. Therefore, it is suggested that ITIH1 and ITIH4 are involved in the stabilization of the ECM^29^. Thus, they accumulate in vascular endothelium and might have a role in the stabilization of endothelial cells and/or ECM damaged by high-altitude hypoxia. Low expression of ITIH4 347–356 (Peak 1, m/z: 1061.91) and ITIH1 205–214 (Peak 2, m/z: 1088.33) in HHI serum samples at sea level may play a significant role in the pathology of HHI by affecting the conditions of vascular endothelial cells. Our study showed that high expression of ITIH4 347–356 (Peak 1, m/z: 1061.91) and ITIH1 205–214 (Peak 2, m/z: 1088.33) in HHA samples may aid adaptation to high-altitude hypoxia.

The third identified serum biomarker for HHA at sea level, FGA 588–624 (Peak 3, m/z: 4057.73), was down-regulated in HHA samples at sea level, and its full protein FGA was also tested by ELISA for its expression in the HHI and HHA groups. ELISA also showed the same tendency as the peptides identified, although there were differences between the expression levels of peptide FGA 588–624 and FGA. Fibrinogen is a plasma glycoprotein that participates in the final phase of blood coagulation. During the last decade, numerous epidemiological studies have demonstrated that increased circulating levels of fibrinogen are a major risk factor for cardiovascular disease, and fibrinogen biosynthesis is up-regulated during the acute phase response^30^,^31^. In this study, higher expression level of FGA 588–624 in HHI samples at sea level may induce blood coagulation enhancement, thus lead to subjects of HHI could not adapt to high-altitude hypoxia environments.

In conclusion, this study aimed to reveal serum peptide biomarkers for people who can adapt to high altitude. We found that hemoglobin concentration and RBC were significantly higher in HHI than HHA samples at high altitude. Serum peptidome also generated differentiated expression peaks between HHI and HHA samples at sea level. Three identified peptides ITIH4 347–356 (Peak 1, m/z: 1061.91), ITIH1 205–214 (Peak 2, m/z: 1088.33), and FGA 588–624 (Peak 3, m/z: 4057.73) were considered to be serum biomarkers for HHA. The method used in this study could provide a new approach to identify potential biomarkers for screening people at sea level who can adapt to high altitude. This is believed to be the first study of serum biomarkers for screening people who can adapt to high-altitude hypoxia. The current study had a limited sample number and was male only; therefore, further study is needed in a large cohort of male and female individuals.

## Materials and Methods

### Human subjects and sample collection

Two hundred healthy men (all Chinese Han; aged 20–25 years) who resided principally at an elevation of 200 meters or lower were recruited between March and June 2013. None of the study population had any previous history of risk factors of HHI, and prior to the present study, they had not been exposed to high altitude. Serum samples from these young men were firstly collected at sea level. Then, they were sent to high altitude at Linzhi (Tibet) with an average elevation of more than 3,000 meters. After 7 days adaption to high altitude, 48 subjects had their diagnosis of acute mountain sickness confirmed by a Lake Louise score ≥ 3 with (including a headache score ≥ 1). These 48 subjects could not adapt to high altitude and formed the HHI group. For comparative approach, another 48 subjects were selected randomly from 152 subjects, which with a lake Louise scores < 3 and without a headache, were selected to form the HHA group. Plasma samples of HHA and HHI groups for routine blood tests at high altitude were collected at the people’s hospital of Linzhi (Tibet).

Approval for this research was obtained from the Ethics Committee and the Human Research Review Committee of Xi’an Jiaotong University (Xi’an, China). All participants underwent medical screening, and written informed consent was obtained after the possible risks of the study were explained. All experiments were carried out in accordance with the approved guidelines.

### Routine blood tests

All the blood samples from the HHA and HHI groups, which collected after 7 days at high altitude as well as which collected at sea level, were subjected to routine testing for hemoglobin, red blood cell count (RBC), packed-cell volume (PCV), mean corpuscular volume (MCV), mean corpuscular hemoglobin (MCH), white blood cell count (WBC), lymphocyte count (LYM), MID (middle cell), polymorphonuclear neutrophil count (PMN), platelet count (PLT), mean corpuscular hemoglobin concentration (MCHC), lymphocyte proportion (LYMPH), MXD (the percentage of median cells), NEUT (neutrophil granulocyte percent), red blood cell distribution width variable coefficient (RDW-CV), red blood cell distribution width standard deviation (RDW-SD), mean platelet volume (MPV), platelet distribution width (PDW), plateletcrit (PCT) and platelet-large cell rate (P-LCR). All the routine blood test results were statistically analyzed by PRISM software.

### Serum samples of HHA and HHI groups at sea level

For serum peptidome and ELISA, serum samples were collected from the HHA and HHI groups at sea level. All blood samples were obtained from non-fasting people. The serum samples were collected in 10 mL separator tubes and kept at 4 °C for 1 h, then centrifuged at 3,000 rpm for 20 min at 4 °C. The serum samples were distributed into 200 μ L aliquots and stored at − 80 °C until use.

### MS analysis: weak cation-exchange chromatography fractionation and MALDI-TOF MS

We used magnetic-bead-based weak cation - exchange chromatography (MB- WCX) (ClinProt purification reagent sets; Bruker Daltonics, Bremen, Germany) for peptidome separation of all serum samples. The 96 serum samples were fractionated according to the standard protocol. With the magnet lowered, 5 μ L serum samples were diluted in 10 μ L binding solution in a standard thin wall PCR tube, added to 10 μ L of MB-WCX beads and then carefully mixed using the mixing feature of the robot. After thorough stirring, samples were incubated at room temperature for 5 min, then the tubes were placed into the magnetic separator to collect the beads on the wall of the tube until the supernatant was clear (~1 min). The supernatant was then removed and the magnet was lowered again. Following the stepwise application of sample and MB-WCX separation, we eluted the peptide fraction from the magnetic beads with 5 μ L of elution solution and 5 μ L of stabilization buffer. The eluted peptides were spotted onto the MALDI AnchorChip with 1 μ L α - cyano - 4 -hydroxy -cinnamic acid (Bruker) in 50% acetonitrile, and 0.5% trifluoroacetic acid was added twice to the MALDI AnchorChip surface. Samples were spotted in triplicate to evaluate the reproducibility of this method.

### Data analysis with ClinProTools

Targets were tested immediately by a calibrated Autoflex III MALDI-TOF MS (Bruker), using FlexControl version 3.0 software (Bruker) in an optimized measuring protocol. A standard calibration mixture of peptides and proteins (mass range: 1–10 kDa) was used for mass calibration. All tests were performed in a blinded manner, including serum analysis of different groups. Data analysis was performed by FlexAnalysis version 3.0 software (Bruker). Recognition of peptide patterns was analyzed by ClinProTools version 2.2 software (Bruker). Data were processed by a standard workflow, which comprised spectral pretreatment, peak selection, and peak calculation.

Genetic Algorithm (GA) mathematical algorithms were used for model analysis. GA is based on evolutionary survival. The best peak clusters were combined into a new feature and the poor clusters were removed, and this process was iteratively repeated until the optimal peak combination was identified. GA works on a population, which consists of a multitude of peak combinations. During selection, the fittest peak combinations are chosen and the less capable are abandoned.

### Peptide identification by LC-ESI-MS/MS

After completing the statistical analysis, selected peptides or proteins biomarkers were further purified and separated by Nano Aquity UPLC C18 beads and serially eluted with 5% and 95% acetonitrile. These proteins and peptides biomarkers were identified directly by a nano-liquid chromatography- electrospray ionization-tandem mass spectrometry system (LC-ESI-MS/MS) consisting of an Acquity UPLC system and an LTQ Orbitrap XL mass spectrometer (Thermo Fisher) equipped with a Nano-ESI source. The settings of the Nano Ion Source are as follows: spray voltage, 1.8 kV; MS scan time, 60 min; and scanning range, m/z 400 to 2000. Obitrap was used for the first scan (MS), with a resolution of 100000, and LTQ was used for the CID and the second scan (MS/MS). The acquired data were searched against the UniProt protein sequence database of HUMAN (http://www.uniprot.org).

### ELISA

All serum samples were analyzed in a blinded fashion, standards and samples were run in triplicate. The concentrations of inter-α trypsin inhibitor heavy chain H1 fragment (ITIH1), inter-α trypsin inhibitor heavy chain H4 fragment (ITIH4) and isoform 1 of fibrinogen α chain precursor (FGA) were respectively quantified using a Human ITIH1 ELISA Kit (No. ab114721), Human ITIH4 ELISA Kit (No. Hu50130) and Human FGA ELISA Kit (No. ab171578). A standard curve was generated and used to determine the concentrations of ITIH1, ITIH4 and FGA in the samples analyzed.

### Statistical analysis

Statistical analysis was performed using GraphPad Prism version 5.0 (GraphPad Software, La Jolla, CA, USA). All data were shown as the mean ±  SD. P <  0.05 was considered statistically significant. Comparisons between HHA and HHI were performed using non-parametric *t* test in routine blood test analysis and ELISA.

## Additional Information

**How to cite this article**: Yang, J. *et al.* Identification of novel serum peptide biomarkers for high-altitude adaptation: a comparative approach. *Sci. Rep.* **6**, 25489; doi: 10.1038/srep25489 (2016).

## Supplementary Material

Supplementary Information

## Figures and Tables

**Figure 1:**
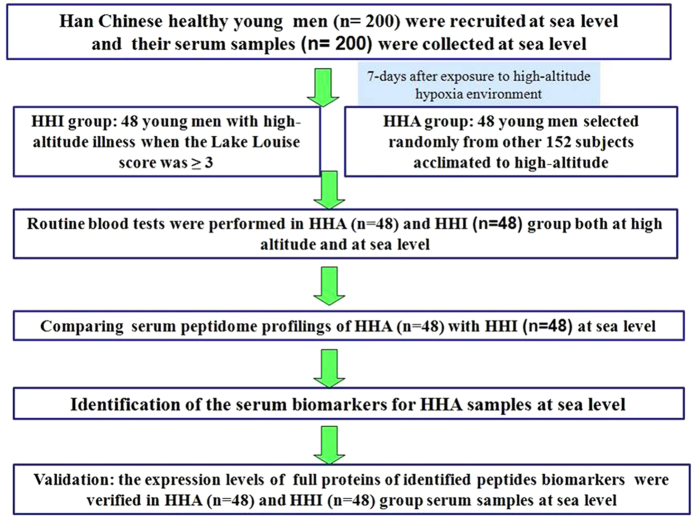
A flow chat for the identification of serum biomarkers for HHA at sea level.

**Table 1:** Results of routine blood tests in HHA and HHI groups at high altitude.

Tests	HHA	HHI	P value
Hemoglobin (g/L)	129.1 ± 1.60	138.5 ± 1.67	P = 0.002
RBC (10^12^/L)	4.47 ± 0.04	4.96 ± 0.08	P = 0.002
PCV (%)	43.99 ± 0.68	45.28 ± 0.96	P > 0.05
MCV	98.32 ± 2.31	98.13 ± 1.33	P > 0.05
MCH (Pg)	28.74 ± 1.84	29.43 ± 0.96	P > 0.05
WBC (10^9^/L)	6.09 ± 0.73	6.01 ± 0.81	P > 0.05
LYM (10^9^/L)	2.03 ± 0.53	1.94 ± 0.38	P > 0.05
PMN (10^9^/L)	3.71 ± 0.88	3.67 ± 0.74	P > 0.05
MCHC (g/L)	315.42 ± 26.44	326.17 ± 7.42	P > 0.05
LYMPH (%)	29.24 ± 4.81	29.56 ± 3.83	P > 0.05
RDW-CV (%)	13.51 ± 0.91	13.45 ± 0.52	P > 0.05
RDW-SD	46.12 ± 6.59	45.23 ± 7.23	P > 0.05
PLT (10^9^/L)	222.50 ± 50.35	218.21 ± 50.24	P > 0.05
MPV	10.58 ± 0.76	10.88 ± 0.77	P > 0.05
PDW	14.85 ± 2.33	19.68 ± 3.27	P > 0.05
PCT (%)	0.19 ± 0.05	0.19 ± 0.04	P > 0.05
P-LCR	33.95 ± 5.96	32.85 ± 4.03	P > 0.05

**Table 2:** Mean levels of ten differentially expressed proteins in HHA and HHI samples collected at sea level.

Peaks (m/z, Da)	P value	HHA	HHI	Fold
1088.33	P < 0.00001	6.98 ± 1.59	2.61 ± 0.68	2.67
4057.63	**P < 0.00001**	**6.15 ± 1.57**	**14.69 ± 3.71**	**0.42**
1061.91	P < 0.00001	38.50 ± 6.46	12.85 ± 4.24	2.99
1115.81	P < 0.00001	3.70 ± 0.46	1.84 ± 0.31	2.01
1999.74	P < 0.00001	2.67 ± 0.37	1.29 ± 0.21	2.07
4969.09	**P < 0.00001**	**3.62 ± 0.59**	**7.79 ± 1.23**	**0.46**
1360.60	P < 0.00001	3.53 ± 0.87	1.02 ± 0.19	3.46
2155.86	P < 0.00001	3.30 ± 0.89	1.23 ± 0.38	2.68
3063.52	P < 0.00001	4.89 ± 1.96	1.57 ± 1.01	3.11
2865.17	P < 0.00001	6.16 ± 1.37	2.98 ± 0.86	2.07

**Table 3:** Sequence identification of three serum peptide biomarkers for HHA at sea level.

Mass (Da) m/z	Peptide regions	Uniprot ID	Peptide sequence	Identity
1061.91	347–356	H7COL5 ITIH4_HUMAN	SEMVVAGKLQ	Inter-α trypsin inhibitor heavy chain H4 (ITIH4) fragment
1088.33	205–214	B7Z539 ITIH1_HUMAN	LDAQASFLPK	Inter-α trypsin inhibitor heavy chain H1 (ITIH1) fragment
4057.73	588–624	P02671 FIBA _HUMAN	SYNRGDSTFESKSYKMADEAGSEADHEGTHSTKRGHA	Isoform 1 of fibrinogen α (FGA) chain precursor

**Table 4:** Serum expression levels of ITIH1, ITIH4 and FGA in HHA and HHI groups.

	HHA	HHI	P value
ITIH1 (range) ng/mL	3.69–5.00	3.32–4.37	0.003
Mean ± Std	4.49 ± 0.12	3.61 ± 0.09	
ITIH4 (range) ng/mL	4.82–6.64	4.38–5.65	0.003
Mean ± Std	5.67 ± 0.17	4.92 ± 0.12	
FGA (range) ng/mL	338.5–471.0	395.6–493.8	> 0.05
Mean ± Std	400.5 ± 13.95	446.6 ± 1.23	
