# Supplementary material for: Identification of novel serum peptide biomarkers for high-altitude adaptation: a comparative approach
Source: Sci Rep. 2016 May 6;6:25489. doi: 10.1038/srep25489 (PMC4858689; doi:10.1038/srep25489)
Supplement: Supplementary Information [file srep25489-s1.doc]

**Manuscript title**:

**Identification of novel serum peptide biomarkers for high-altitude adaptation: a comparative approach**

Juan Yang **^1,2,+^**, Wenhua Li **^3,+^** , Siyuan Liu **^1,2^**, Dongya Yuan **^3^**, Yijiao Guo**^3^**, Cheng Jia **^3^**, Tusheng Song **^1,2^** & Chen Huang **^1,2,*^**

Figure S1, MS/MS identification of peak 1088.33 Da

Figure S2, MS/MS identification of peak 1061.91 Da

Figure S3 MS/MS identification of peak 4057.63 Da

**Table S1. Results of routine blood tests in HHA and HHI groups at sea level**

| **Tests** | **HHA** | **HHI** | **P value** |
| --- | --- | --- | --- |
| Hemoglobin (g/L) | 107.7 ± 5.83 | 107.8 ±5.13 | P > 0.05 |
| RBC (10^12^/L) | 4.54± 0.34 | 4.92± 0.08 | P > 0.05 |
| PCV (%) | 45.56± 4.08 | 44.91± 4.29 | P > 0.05 |
| MCV | 98.23± 1.65 | 97.96± 2.61 | P > 0.05 |
| MCH (Pg) | 29.59±1.02 | 29.31± 1.46 | P > 0.05 |
| WBC (10^9^/L) | 6.07±0.93 | 6.21±0.96 | P > 0.05 |
| LYM (10^9^/L) | 1.93 ± 0.40 | 1.95 ±0.43 | P > 0.05 |
| PMN (10^9^/L) | 3.91 ±1.08 | 3.83 ± 0.96 | P > 0.05 |
| MCHC (g/L) | 323.76 ± 10.65 | 321.79 ± 22.12 | P > 0.05 |
| LYMPH (%) | 29.83 ±3.89 | 29.76 ± 5.24 | P > 0.05 |
| RDW-CV (%) | 13.56 ± 0.57 | 13.35±0.84 | P > 0.05 |
| RDW-SD | 45.56 ± 4.08 | 44.90 ± 4.29 | P > 0.05 |
| PLT (10^9^/L) | 212.66±54.50 | 212.11±44.52 | P > 0.05 |
| MPV | 10.75 ± 0.63 | 10.68 ± 1.28 | P > 0.05 |
| PDW | 14.67±1.16 | 14.69±1.65 | P > 0.05 |
| PCT(%) | 0.17±0.04 | 0.19±0.04 | P > 0.05 |
| P-LCR | 33.37±4.83 | 33.162±4.82 | P > 0.05 |
